# Snail Mucus Protective Effect on Ethanol-Induced Gastric Ulcers in Mice

**DOI:** 10.3390/life12081106

**Published:** 2022-07-22

**Authors:** Lubomir Petrov, Mihail Kachaunov, Albena Alexandrova, Elina Tsvetanova, Almira Georgieva, Aleksander Dolashki, Lyudmila Velkova, Pavlina Dolashka

**Affiliations:** 1 National Sports Academy “Vassil Levski”, 23, Acad. Stefan Mladenov Str., Studentski Grad, 1700 Sofia, Bulgaria; dr.lubomir.petrov@gmail.com (L.P.); mihailkachaunov@gmail.com (M.K.); 2 Laboratory of Free Radical Processes, Institute of Neurobiology, Bulgarian Academy of Sciences, 23, Acad. Georgi Bonchev Str., 1113 Sofia, Bulgaria; elinanesta@abv.bg (E.T.); almirageorgieva@gmail.com (A.G.); 3 Institute of Organic Chemistry with Centre of Phytochemistry, Bulgarian Academy of Sciences, 9, Acad. Georgi Bonchev Str., 1113 Sofia, Bulgaria; aleksandar.dolashki@orgchm.bas.bg (A.D.); lyudmila_velkova@abv.bg (L.V.); pda54@abv.bg (P.D.)

**Keywords:** gastric ulcers, *Cornu aspersum* mucus, oxidative stress

## Abstract

Nowadays, an increased interest in natural compounds with preventive or therapeutic potential for various diseases has been observed. Given the involvement of oxidative stress in the pathogenesis of gastric ulcer (GU) and the wide range of bioactive compounds isolated from snails, this study aimed to investigate the protective effect of *Cornu aspersum* (Müller, 1774) mucus on ethanol-induced GUs. Male albino mice were divided into Control, Ethanol, Mucus + Ethanol and Mucus + Omeprazole treated groups. The GUs were induced by administration of 96% ethanol (10 mL/kg, per os). One hour before ulcer induction, the mice of Mucus + Ethanol group were pretreated with mucus (20 mg/kg, per os), and the mice of Mucus + Omeprazole group were pretreated with omeprazole (20 mg/kg, per os). Ethanol administration caused grave lesions of gastric mucosa and a significant decrease of glutathione (GSH) and superoxide dismutase (SOD), catalase, and glutathione reductase (GR) activities. In the animals with mucus or omeprazole pre-administration compared to the Ethanol group, the following were observed: only a small number of hemorrhagic fields, significantly reduced GU index with calculated 73% protection by mucus and 78% protection by omeprazole, and significant recovery of mucosal GSH and SOD and GR activities. In addition, the mucus inhibited *Helicobacter pylori* growth. Thus, the protective effect of *C. aspersum* mucus on both gastric mucosa and gastric antioxidant potential in ethanol-induced GU model suggests that it may serve as a good tool for prevention of this disease.

## 1. Introduction

Digestive system diseases, such as functional gastrointestinal disorders, gastritis, and peptic ulcers, are ubiquitous worldwide. Although not life threatening in general, these pathological conditions can significantly impair the quality of affected people’s life and have a broad negative socioeconomic effect [1]. Moreover, it has been found that gastric ulcer patients are at higher risk of developing gastric cancer [2,3].

Gastric ulcer (GU) disease affects about 10% of the population [1,4]. Several classes of drugs are used in GU treatment: proton pump inhibitors, M1-receptor blockers, and H2-receptor antagonists. However, many side effects are associated with these drugs, such as arrhythmia, hematopoietic changes, erectile dysfunction, and gynecomastia [5]. Therefore, new drugs with less side effects than those currently used are needed.

The regenerative capacity of snail mucus against their own shell and skin injuries attracted human attention, and it has been traditionally used therapeutically against gastrointestinal ulcers since ancient Greece [6,7]. Nowadays, studies have demonstrated the protective effect of mucus from the giant African snail in different models of gastric ulcers: induced by indomethacin, histamine, and prolonged exposure of the experimental mice to cold [8,9]. The results show a pronounced beneficial effect, likely due to the cytoprotective and antispasmodic action of the mucus.

Acute gastric ulcers result most frequently from *H. pylori* infection, alcohol consumption, and non-steroidal anti-inflammatory drugs intake, as well as from psychological and physical stress. All of these stressors may induce oxidative stress that is able to initiate and aggravate gastric disorders [10,11]. Oxidative stress (OS) is a condition that is characterized by an imbalance between pro-oxidant processes induced by reactive oxygen species (ROS) and the capacity of the antioxidant protection of organisms to prevent their excess production or overcome the consequences of their action. Much data has shown that increased ROS formation is not only a trigger of inflammation, but the inflammation itself provokes ROS production in gastric disorders [12,13]. In addition, peptic ulcers and other phenotypes of gastrointestinal diseases (e.g., gastroparesis) are known to be related to antioxidant property dysfunction [10].

It has been found that mucus, isolated from the common garden snail *C.* *aspersum* (synonym *Helix aspersa*), comprises various substances with different bioactive properties with antioxidant potential [14,15,16]. Therefore, given the involvement of OS in the pathogenesis of GU, as well as the wide range of bioactive compounds that underlies the broad pharmacological activities of *C. aspersum* mucus, this study aimed to investigate the possible protective effect of *C. aspersum* mucus on ethanol-induced GUs in mice and elucidate its efficacy mechanism in terms of OS measures.

## 2. Materials and Methods

### 2.1. Snail Mucus Collection

Crude mucus was collected from *C. aspersum* snails grown in Bulgarian eco-farms using patented technology [17,18] without causing suffering in any snail. In brief, the snails were placed in a special device, where electrical stimulation with low voltage caused snails to release mucus without compromising their biological function. After treatment, the snails were returned to the farm. The obtained fresh extract was homogenized and subjected to centrifugation to remove coarse impurities. The purification of supernatant included several steps of filtration, using filters with smaller pore sizes for each subsequent filtration [17,18,19]. The 2 mucus fractions with molecular weights below 10 and below 20 kDa were obtained by using ultrafiltration membranes, at 4 °C, as follows: discs from Ultracel^®^ Regenerated Cellulose with pore sizes from 10 kDa NMW, (EMD Millipore Corporation, Billerica, MA, USA) and polyethersulfone membrane with pore sizes from 20 kDa (Microdyn Nadir™ from STERLITECH Corporation, Auburn, AL, USA) [18]. Samples were filtered through a syringe filter (Millipore, Millex-HV, 0.22 µm pore size, hydrophilic PVDF membrane) for removing microorganisms. The purified mucus extracts were stored at 4 °C, as previously reported by Trapella et al., 2018 [16]. Thus, to obtain mucus extract and two peptide fractions, non-invasive techniques—a series of centrifugation and filtrations, as well as ultrafiltration, which ensure the production of intact peptides and proteins—were used.

### 2.2. SDS-PAGE Electrophoresis

The native fresh extract from mucus was analyzed by sodium dodecyl sulfate-polyacrylamide gel electrophoresis (SDS-PAGE) using 5% stacking gel and 12.5% resolving gel according to the Laemmli method [20]. About 20 μg of the sample was loaded on the gel. Protein standard mixture ranging from 10 kDa to 250 kDa (Precision Plus Protein™ Standard All Blue, Bio-Rad Laboratories, Munich, Germany) was used as a molecular marker. Coomassie Brilliant Blue G-250 staining was used for the visualization.

### 2.3. MALDI-TOF-MS

The isolated mucus extract was lyophilized and analyzed by MALDI-TOF-TOF mass spectrometry on an AutoflexTM III High-Performance MALDI-TOF & TOF/TOF System (Bruker Daltonics, Bremen, Germany), which uses a 200 Hz frequency-tripled Nd–YAG laser operating at a wavelength of 355 nm. Analysis was carried out after mixing 2.0 μL of the sample with 2.0 μL of matrix solution (7 mg/mL of 3,5-dimethoxy-4-hydroxycinnamic acid (Sinapinic acid)) in 50% CAN containing 0.1% TFA), but only 1.0 μL of the mixture was spotted on a stainless steel 192-well target plate. The samples were allowed to dry at room temperature before being analyzed. A total of 3500 shots were acquired in the MS mode, and collision energy of 4200 was applied. All spectra were obtained using the positive-ion mode. Standard stainless-steel targets obtained from the manufacturer were employed for all analyses. The spectra in this study were calibrated using instrumental calibration based on the parameters determined from the analysis of standard proteins and peptides.

### 2.4. Antibacterial Activity of Mucus Extracts against H. pylori

The susceptibility of *H. pylori* to mucus extracts was determined by the agar well diffusion method [21]. Three fractions isolated from *C. aspersum* mucus: Fraction 1—native mucus extract (0.5 mg/mL), Fraction 2—mucus containing components with molecular weight (MW) < 20 kDa (0.48 mg/mL), and Fraction 3—mucus containing components MW < 10 kDa (0.45 mg/mL) have been analyzed. The samples were qualitatively tested according to the growth inhibition assay.

Sterile molten Mueller–Hinton agar at 45 °C, supplemented with 5% defibrinated horse blood, was prepared and mixed with three concentrations (0.25, 0.75, and 1.0 mL per 20 mL agar) of native mucus extract. The mixture was aseptically poured into Petri dishes and allowed to set. A sterile cork-borer with a diameter of 3 mm was used to make equidistant wells in the agar. A hundred microliters (100 µL) of a fresh overnight culture of *H. pylori* was poured into the wells. The prepared samples were incubated in a microaerophilic incubator at 37 ± 1 °C for 24–48 h. The plates were observed for inhibition, and the diameter zones of inhibition were measured to the nearest millimeter. All tests were carried out in three repetitions to ensure accuracy

### 2.5. Animals

A total of 30 male albino mice, weighing 25–35 g (10 weeks of age), were randomly divided into 4 groups of 10 animals each: Control (untreated mice), Ethanol treated (mice with ethanol-induced gastric ulcers), Mucus + Ethanol treated (mice pretreated with snail mucus and ethanol-induced gastric ulcers), and Omeprazole + Ethanol treated (mice pretreated with omeprazole and ethanol-induced gastric ulcers). Each group was kept in a separate cage in a controlled temperature room (24 ± 2 °C) with a 12 h light/dark cycle. The animals had free access to food and water. The mice were maintained and used in accordance with the guidelines of the Care and Use of Laboratory Animals (US National Institute of Health) and the rules of the Ethics Committee of the Institute of Neurobiology, Bulgarian Academy of Sciences (registration FWA 00003059 by the US Department of Health and Human Services).

### 2.6. Ulcer Damages

Twenty-four hours prior to the experiment, the feeding of mice was withdrawn; however, the animals had free access to water. One hour before ulcer induction, the mice of the Mucus + Ethanol group were pretreated with snail mucus (20 mg/kg, per os), and the mice of the Omeprazole + Ethanol group were pretreated with omeprazole (20 mg/kg, per os). Gastric ulcers were induced by the administration of 96% ethanol (10 mL/kg, per os) in Ethanol, Mucus + Ethanol, and Omeprazole + Ethanol groups. One hour later, mice from all four groups were euthanized by cervical dislocation under pentobarbital (40 mg/kg, intraperitoneal injection) anesthesia. The stomachs were excised, opened over the greater curvature, and rinsed with saline solution (0.9%). Pictures of stoma mucous were taken using a digital camera; the ulcers were quantified, and the gastric damage area (mm^2^) was determined using the image analysis program Image J [22].

### 2.7. Gastric Ulcer Index

The Gastric Ulcer Index (GUI) for each mouse was calculated according to Ganguly [23]: GUI = (TAGU (mm^2^) × 100)/(TGA (mm^2^)), where TAGU is the total area of gastric ulcers, and TGA is the total gastric area of each mouse. To calculate the protection percentage (PP) [24], the following formula was used: PP = (GUI control − GUI treated)/(GUI control) × 100.

### 2.8. Tissue Preparation

A small portion of each stomach was cut and homogenized in cold potassium phosphate buffer (0.05 M, pH 7.4). The homogenate was centrifuged at 3000 rpm for 10 min. A part of the obtained supernatant was used to determine the lipid peroxidation (LPO) level and glutathione (GSH) concentrations. The other part was additionally centrifuged at 12,000 rpm for 20 min, and the supernatant was used for measurement of the activities of the antioxidant enzymes superoxide dismutase (SOD), catalase (CAT), and glutathione reductase (GR).

### 2.9. Biochemical Analyzes

The OS biomarkers were measured spectrophotometrically using commercially available kits: Lipid Peroxidation (MDA) Assay Kit MAK085, Glutathione Assay Kit CS0260, SOD Assay Kit-WST 19160, Catalase Assay Kit CAT100, and Glutathione Reductase Cellular Activity Assay CRSA (Sigma-Aldrich Co., LLC., St. Louis, MO, USA). The manufacturer’s working instructions were strictly followed.

### 2.10. Statistics

Descriptive statistics, the Shapiro–Wilks test of normality and One-Way ANOVA with Tukey post hoc test were applied using the statistical program GraphPad Prism 7.0. In text, all data are presented as the mean ± standard deviation (SD) and in the figures as the mean ± standard error of measurement (SEM).

## 3. Results

### 3.1. Composition of C. aspersum Mucus

**Figure 1:**
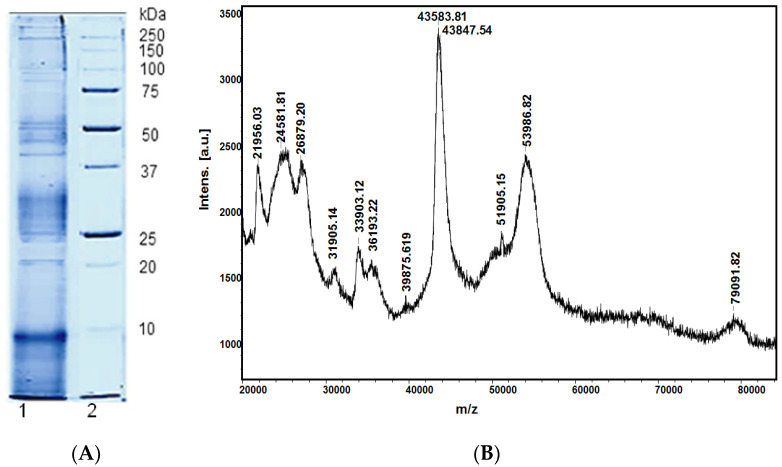
(**A**) 12.5% SDS–PAGE with Coomassie Brilliant Blue G-250 staining of: 1, crud extract of *C. aspersum* mucus; and 2, standard protein marker (Protein Prestained Standards, Biorad); (**B**) MALDI-TOF-MS spectrum of *C. aspersum* mucus, recorded between 20–80 kDa.

The analysis by 12.5% SDS-PAGE showed that the mucus is a complex mixture of substances with different molecular weights. Several protein bands in the range of 25–35 kDa, 38–40 kDa, 45–50 kDa; 80–90 kDa, and above 250 kDa were detected by SDS-PAGE (Figure 1A). Moreover, by MALDI-MS analysis in the region of 20–80 kDa, the exact molecular masses of mucus proteins were determined (Figure 1B). In the region above 100 kDa, proteins might correspond to glycoproteins and mucins. More details on the *C. aspersum* mucus composition based on our earlier studies [15,18,19,25,26,27] are presented in Table 1. A number of new peptides have been isolated from the mucus, and their amino acid sequences were determined by de novo sequencing and by MALDI-TOF-MS/MS analyses; some of them are presented in Table 1. Most of the peptides with MW < 3 kDa were characterized by an amphipathic structure, had a positive net charge, pI < 7.0, and displayed generally hydrophobic surfaces (GRAVY > 0) (Table 1).

### 3.2. In Vitro Susceptibility of H. pylori to Native Mucus Extract

The antibacterial efficacy of mucus extracts from garden snail *C. aspersum* against bacterial strain *H. pylori* was identified by agar dilution. The growth of the bacterial strain was monitored at 24 and 48 h after treatment with 0.25 mL, 0.75 mL, and 1.0 mL from Fraction 1—native mucus extract, Fraction 2 with MW < 20 kDa, and Fraction 3 with MW < 10 kDa.

**Figure 2:**
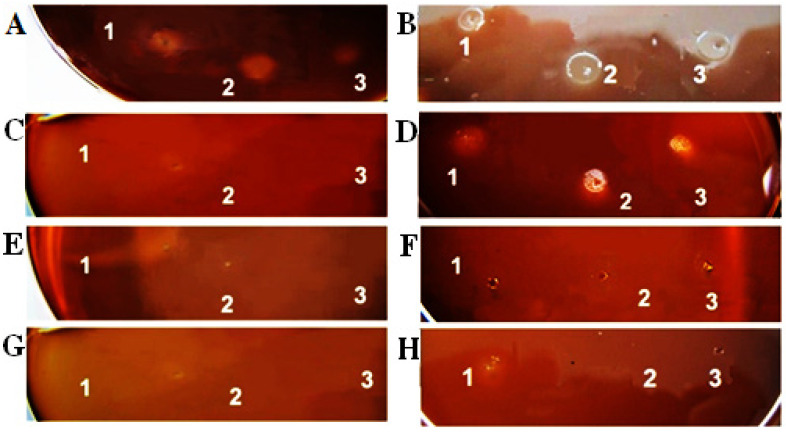
The antibacterial effect on the growth of the bacterial strain *H. pylori* of Fraction 1 in concentrations: (**A**) Control—without extract after 24 h incubation; (**B**) Control—without extract after 48 h incubation; (**C**) 0.25 mL Fraction 1 after 24 h incubation; (**D**) 0.25 mL Fraction 1 after 48 h of incubation; (**E**) 0.75 mL Fraction 1 after 24 h incubation; (**F**) 0.75 mL Fraction 1 after 48 h of incubation; (**G**) 1.0 mL Fraction 1 after 24 h incubation; (**H**) 1.0 mL Fraction 1 after 48 h of incubation.

The obtained results showed that both fractions, Fraction 2 and Fraction 3, did not affect the growth of *H. pylori*. As shown in Figure 2, only native mucus extract inhibited bacterial growth at 24 and 48 h. *H. pylori* growth was observed in the control sample without extract after 24 h (Figure 2A) and reached its maximum size at 48 h of incubation, with a spot diameter of 10.5 mm (Figure 2B).

Testing of Fraction 1 with the lowest concentration (0.25 mL per 20 mL agar) showed significant inhibition of bacterial growth at 24 h (Figure 2C) as opposed to 48 h (Figure 2D). Despite the observed bacterial growth at 48 h, the growth zone decreased from 10.5 mm in diameter to 8.0 mm compared to the control. Antibacterial tests performed with higher concentrations, 0.75 mL and 1.0 mL per 20 mL agar of the natural mucus extract, showed inhibition of bacterial growth at both 24 (Figure 2E,G, respectively) and 48 (Figure 2F,H, respectively) h. Application of Fraction 1 with the highest concentration resulted in the most significant anti-*H. pylori* effect.

Since the application of native mucus extract resulted in the most significant anti-H. pylori effect, the in vivo studies were performed only with it.

### 3.3. Effect of C. aspersum Mucus on Ethanol-Induced Gastric Ulcers

**Figure 3:**
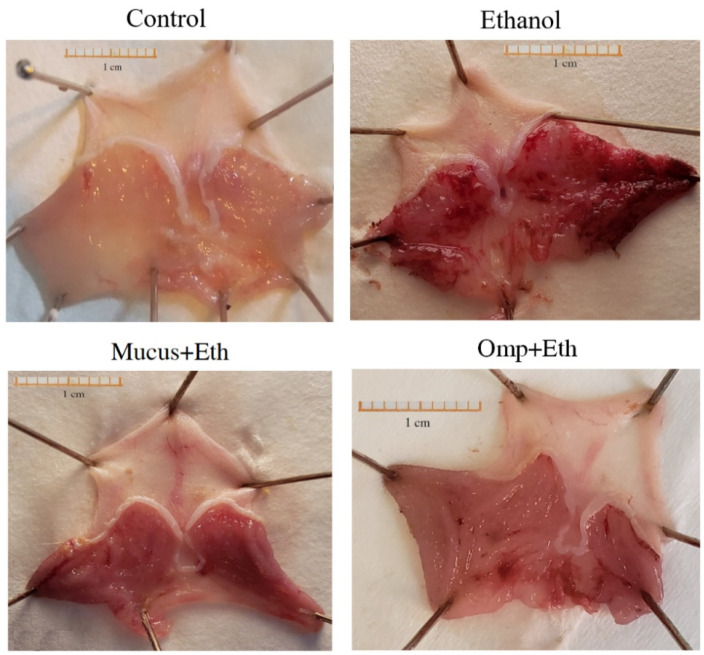
Effect of snail mucus and omeprazole on the macroscopic morphology in mice with ethanol-induced gastric ulcers.

As shown in Figure 3, the animals that had received ethanol (10 mL/kg; 96%) had grave lesions with large hemorrhagic necrosis of the gastric mucosa. In the animals with pre-administration of snail mucus (20 mg/kg) or omeprazole (20 mg/kg), only a small number of hemorrhagic fields were observed.

**Figure 4:**
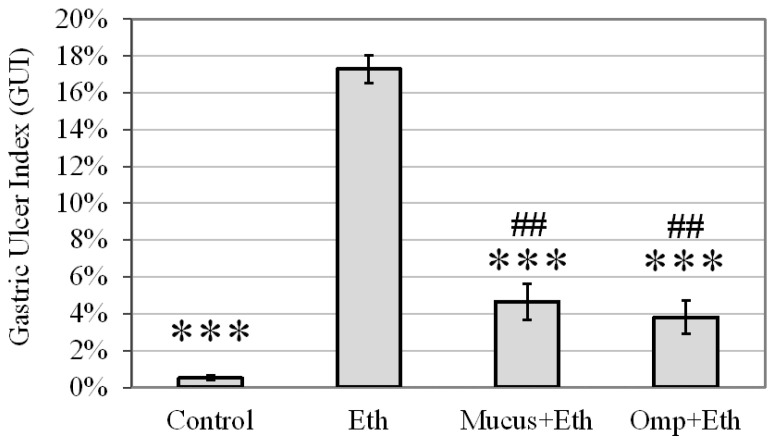
Average Gastric Ulcer Index (GUI) in the four experimental groups: Control, Ethanol (Eth), Mucus + Ethanol (Mucus + Eth), and Omeprazole + Ethanol (Omp + Eth); ***—significance *p* < 0.001 vs. Ethanol group, ##—significance *p* < 0.01 vs. Control group.

The attenuation of the hemorrhagic mucosal lesions in the snail-mucus-pretreated mice was notable in comparison with the Ethanol group. Figure 4 shows that GUI was significantly reduced in the mice pretreated with snail mucus compared to the Ethanol group (4.7% vs. 17.3%, respectively) (*p* < 0.001). The protection percentage (PP) was calculated to be 73%. The effect of snail mucus was similar to those of omeprazole in which PP was calculated to be 78%.

### 3.4. Effect of C. aspersum Mucus on Oxidative Stress Parameters

**Figure 5:**
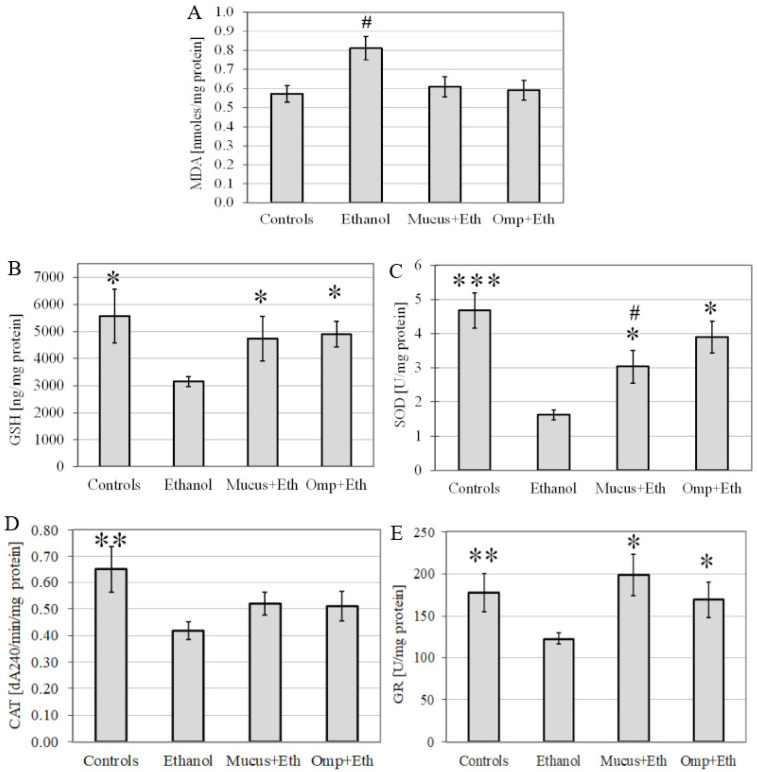
Lipid peroxidation level (**A**); GSH concentration (**B**); and antioxidant enzymes activity of SOD (**C**); CAT (**D**); and GR (**E**); in stomach homogenate from the four experimental groups: Control, Ethanol, Mucus + Ethanol (Mucus + Eth) and Omeprazole + Ethanol (Omp + Eth); *—significance *p* < 0.05 vs. Ethanol group; ** significance—*p* < 0.01 vs. Ethanol group; ***—significance *p* < 0.001 vs. Ethanol group, #—*p* < 0.05 vs. Control group.

The level of MDA (as an LPO index), the concentration of the GSH, and the activity of antioxidant enzymes (SOD, CAT, and GR) in gastric tissue were evaluated (Figure 5). Gastric MDA was significantly higher in the Ethanol group versus the Control, and the pretreatment with snail mucus led to a decreased MDA concentration. Significant depletion of the GSH concentration in the Ethanol group was observed, and the administration of snail mucus led to the prevention of a decrease in its amount. The activities of the antioxidant enzymes SOD, CAT, and GR in the Ethanol group significantly decreased compared to the Control group. The pretreatment of mice with snail mucus significantly protected SOD and GR activity in relation to the Ethanol group, and CAT activity was slightly (insignificantly) raised. The effect of snail mucus pretreatment was similar to those of omeprazole in terms of all tested parameters of the oxidative status of stomach homogenate of mice with ethanol-induced ulcers.

## 4. Discussion

The ethanol-induced GU animal model is a classical method used for screening compounds that possess anti-ulcer activity. The effect of ethanol on gastric mucosa is multifactorial and complicated. Ethanol exfoliates gastric cells and eventually provokes bleeding. The macroscopic observations in this study showed clearly visible bloody lesions after ethanol treatment. These cause disruption of the mucosal barrier integrity and an increase in the mucosal permeability. This model proves that a strong inflammatory response is associated with augmented neutrophil infiltration and myeloperoxidase activation [28], modulation of the NO signaling pathways [29], and perturbation of inflammatory/anti-inflammatory cytokine balance [30]. All these events trigger excess ROS production and cause oxidative damage with cellular deleterious effects [31,32]. The results obtained in this study are in agreement with the published data for increased levels of LPO, reduced GSH concentration, and decreased antioxidant enzymes’ activities after ethanol administration [33,34]. In this study, the pretreatment of mice with snail mucus led to a notable gastric lesion reduction along with significant preservation of antioxidant potential, probably due to the suppression of OS development after ulcer induction. The effects of snail mucus were comparable to pretreatment with omeprazole, a proton pump inhibitor, used as a positive control in this study. Omeprazole is a widely used drug for GU treatment and has been applied in numerous studies to provide a gastro-protective effect [35,36]. It has been demonstrated that omeprazole has a dual action in gastrointestinal protection by providing potent antioxidant properties in addition to its major role as an acid-suppression agent [37].

Snail mucus is a complex, evolutionary determined multi-component mixture that includes substances that determine its antioxidant properties. The antioxidant activity could be due to amino acid composition and sequence, the steric structure of the peptides and proteins, glycation of the proteins, and the presence of enzymatic and non-enzymatic antioxidants in snail mucus. The peptide and protein bands detected by SDS-PAGE in this research are in accordance with previously obtained results [18,38,39]. In the MW < 10 kD fraction isolated from the mucus of *C. aspersum*, the amino acid sequences of peptides (Table 1) clearly demonstrate the predominant presence of amino acid residues, such as glycine, proline, leucine, valine, tryptophan, aspartic acid, phenylalanine, and arginine, refs. [40,41] some of which are associated with antioxidant activity [15].

A key factor in the ability of the peptide to trap radicals is considered to be the high proportion of hydrophobic amino acids compared to hydrophilic amino acids [41]. Research has shown that some peptide features, namely, low molecular mass, the presence of antioxidant amino acids such as tyrosine, phenylalanine, proline, alanine, histidine, and leucine; hydrophobicity amino acids (histidine, tryptophan, phenylalanine, proline, lysine, leucine, and valine); indole/imidazole/pyrrolidine ring; along with the steric structure at the C- and N-termini, play an important role for peptide antioxidant properties [41]. Thus, the presence of peptide structures 3, 7, 9, 12, 13, 17, 18, 21, and 22 in the applied snail mucus can be related to its antioxidant activity. Some of these peptides contain three Pro residues (Table 1). Several publications have shown that the higher proline content in substances of plant [41] and animal [42] origin is associated with higher antioxidant activity. Peptide antioxidant activity is determined not only by amino acid composition, but also by amino acid sequences and interactions between amino acids in polypeptide chains, the steric structure at the C- and N-termini, and molecular mass. The higher content of hydrophobic amino acids in peptides determines their good radical scavenging effect in comparison to those with a higher content of hydrophilic amino acids. Low peptide molecular mass also contributes to a significant antioxidant effect [43]. It was suggested that the amphiphilic nature of peptides enhances the radical-scavenging activities of peptides by increasing their solubility and in this way facilitating the interaction and proton exchanges with radical species. A large portion of the amino acids in snail mucus proteins are glycated. In the region above 100 kDa, proteins might correspond to mucins and glycoproteins [44]. The healthy stomach lining produces protective mucins, and it can be assumed that the mucins contained in the snail mucus complement their effect. Furthermore, it is possible that snail mucus coats the gastric mucosa, building an extra layer that protects against ethanol exposure. Concerning glycoproteins, it has been reported that they have antioxidant properties [45,46,47]. Several studies [45,46] have shown that the addition of purified glycoproteins prevents DNA damage provoked by hydroxyl radicals. However, it is not clarified which part of the glycoproteins—glycan or protein—is responsible for this activity. It has been suggested that there are differences in the radical scavenging ability of glycoproteins that might be attributed to the glycan part or protein part [47].

Antioxidant enzymes such as SOD and CAT, as well as the non-enzymatic antioxidant GSH, detected in the *C. aspersum* mucus [15,18] probably contribute most to its antioxidant potential. SOD and CAT constitute the first line of defense against ROS [48]. The GSH is an essential part of the antioxidant system, able to directly neutralize ROS such as superoxide radicals, lipid peroxyl and hydroxyl radicals, peroxynitrite, and hydrogen peroxide [49]. GSH is involved in the reduction of other antioxidants in the cell such as vitamin C and indirectly vitamin E [50]. In addition, GSH is a mandatory cofactor of glutathione peroxidases (which reduce H_2_O_2_ and organic peroxides) and glutathione transferases (which reduce organic peroxides) [51]. Oxidized peroxiredoxin that directly reduces peroxides and peroxynitrites is also reduced by GSH [49]. Precise kinetic measurements have shown that peroxiredoxin reduces more than 90% of peroxides in cells [52], surpassing catalase and glutathione peroxidase in this function.

The antioxidant properties of snail mucus are only part of its overall beneficial effect on GUs. The identified in snail mucus hyaluronic acid, chondroitin sulfate, hyaluronan, dermatan sulfate, heparin, and heparin sulfate [53] promote dermal regeneration and epidermal proliferation. The presence of components such as collagen, elastin, glycolic acid, and allantoin as well as the mucopolysaccharide components are associated with the protective effect of snail mucus on the gastric mucosa [53]. Allantoin, collagen, elastin, and glycolic acid that promote collagen synthesis are also found in snail mucus [53]. The collagen and elastin contained in the mucus have a structure close to those in human derma and are in a similar ratio. Therefore, these compounds probably also contribute to the good gastro-protective effect of the snail mucus established in this research.

In addition, in this study, we found that the native mucus extract applied in the highest concentration tested manifested a significant anti-*H. pylori* effect that effectively inhibited bacterial growth at 24 and 48 h, whereas both fractions with MW 10 and MW 20 kDa were not effective. Therefore, the presence of low molecular weight metabolites, peptides, and glycopeptides with antimicrobial activity and antioxidant properties do not sufficiently inhibit the growth of *H. pylori*. This fact leads us to the hypothesis that anti-*H. pyloric* activity is due to the synergy between different components found in the mucus such as secondary metabolites, peptides, proteins, and glycoproteins with antimicrobial activity and the specific ratio between them. Trapella et al. [16] also suggested that snail mucus’s potential as a therapeutic agent in wound repair was attributable to the synergistic activity of several molecules. In mucus, such as we have used in the present paper, the primary structures of novel antimicrobial peptides with molecular weights mainly between 1–3 kDa were identified by tandem mass spectrometry (Table 1), and their antibacterial activity was demonstrated [18,26]. The *C. aspersum* mucus contains both cationic and anionic and neutral peptides, but cationic are dominant. That is considered a prerequisite for observed mucus antimicrobial activity—destruction of biological membranes and/or direct cell lysis [18,26]. The antimicrobial activity was predicted based on the identified primary structures using iAMPpred software (online prediction server available at http://cabgrid.res.in:8080/amppred, accessed on 10 June 2022). The presented results (Table 1) showed that some peptides (№ 2, 11, 16, 17, 19, 20, 23–25, and 27 from Table 1) could be predicted to have antimicrobial activity. Most of these peptides have high levels of glycine (Gly) and leucine (Leu) residues as well as one or two proline (Pro) residues and belong to a new class of Gly/Leu-rich antimicrobial peptides [18,27,40]. It is known that cationic antimicrobial peptides kill microbes via mechanisms that predominantly involve interactions between the peptide’s positively charged residues and anionic components of the target cell’s membranes. Moreover, some of the positively charged peptides may penetrate into the cell to bind intracellular molecules that are crucial to the life of the cell [26]. Recently, different peptide and protein fractions from the *C. aspersum* mucus demonstrated antimicrobial and antioxidant activity as well as regenerative properties [18,19,36]. Dolashki et al. [18] found some bioactive components with MW 10–30 kDa exhibited predominant antibacterial activity against *B. laterosporus* and *E. coli*, and the fraction with MW > 20 kDa manifested a promising antibacterial effect against *C. perfringens. H. aspersa* mucus was also effective against three laboratory strains of *P. aeruginosa* [39].

## 5. Conclusions

In conclusion, the positive effect of *C. aspersum* mucus we found in this model of gastric injury is probably the result of the complex action of many factors, including the protection by molecules with antioxidant properties, substances that ensure tissue regeneration, and compounds with antimicrobial effects which may contribute to the beneficial effect of snail mucus in gastric ulcers and make it an appropriate substance for the prevention of stomach diseases. Obviously, further studies using different injury-inducing models are needed to reveal the particular mechanisms and compounds responsible for the snail mucus’s healthy effects.

## Figures and Tables

**Table 1:** Characterization of peptides in the mucus of the garden snail, *C. aspersum*, determined by de novo MALDI-MS/MS sequencing (GRAVY—grand average of hydropathicity index; pI—Isoelectric point).

No	Amino Acid Sequence of Peptides	Exper. Mass [M + H]^+^ Da	Calcul. Monois. Mass. Da	pI *	GRAVY *	Net Charge *	Predicted Activity		
							Antibac. (%)	Antiviral. (%)	Antifungal. (%)
1 ^a^	γ-ECG (glutathione)	308.058	307.08	3.27	−0.47	−1/0	63.0	54.0	45.0
2 ^a^	LGHDVH	677.32	676.33	5.97	−0.383	−1/0	84.0	76	61.0
3 ^a,b^	LLMGPEV	758.41	757.40	4.00	+1.171	−1/0	33.0	30.0	12.0
4 ^b^	QSGKSPGFGL	977.50	976.50	8.75	−0.520	0/+1	64.0	8.9	26.0
5 ^a^	LFSNQLFN	982.50	981.49	5.52	−0.68	0/0	53.0	38.0	43.0
6 ^c^	LLFSGGQFNG	1039.52	1038.51	5.52	+0.420	0/0	74.0	28.0	59.0
7 ^d^	DLTLNGLSPK	1057.58	1056.58	5.84	−0.300	−1/+1	12.1	17.0	19.0
8 ^d^	MPDGALLGGGGD	1059.71	1058.47	3.56	+0.058	−2/0	50.0	52.0	32.0
9 ^b^	LPDSWEPGGGG	1071.56	1070.47	3.67	−0.882	−2/0	7.7	21.0	6.0
10 ^e^	LGDLNAEFAAG	1077.67	1076.51	3.67	+0.409	−2/0	27.0	46.0	30.0
11 ^a^	LGLGNGGAGGGLVGG	1155.61	1154.60	5.52	+0.687	0/0	86.0	50.8	61.0
12 ^b^	YNGFRPGDCY	1191.49	1190.48	5.83	−1.120	−1/+1	43.0	32.0	55.0
13 ^e^	AGVGAGGANPSTYVG	1277.91	1276.60	5.57	+0.260	0/0	25.0	7.5	11.0
14 ^e^	GAACNLEDGSCLGV	1308.81	1307.55	3.67	+0.564	−2/0	58.0	58.0	53.0
15 ^d^	NLVGGSGGGGRGGANPLG	1496.73	1495.75	9.75	- 0.217	0/+1	66.0	33.7	48.2
16 ^d^	GLLGGGGGAGGGGLVGGLLNG	1609.94	1608.86	5.52	+0.776	0/+1	90.0	53.6	65.0
17 ^d^	MGGLLGGVNGGGKGGGGPGAP	1666.83	1665.83	8.50	+0.005	0/+1	78.6	52.0	61.5
18 ^e^	ASKGCGPGSCPPGDTVAGVG	1716.82	1715.76	5.86	+0.005	−1/+1	25.0	12.0	23.0
19 ^f^	LFGGHQGGGLVGGLWRK	1738.99	1737.94	11.0	−0.024	0/+2	75.6	41.0	78.5
20 ^e^	ACSLLLGGGGVGGGKGGGGHAG	1739.02	1737.86	8.27	+0.409	0/+1	83.0	49.0	67.0
21 ^d^	MLLNAKWAPHSTGPPNA	1804.91	1803.91	8.52	−0.400	0/+1	8.5	11.0	6.9
22 ^e^	ACLTPVDHFFAGMPCGGGP	1877.14	1875.81	5.08	+0.542	−1/0	32.0	43.0	20.0
23 ^e^	NGLFGGLGGGGHGGGGKGPGEGGG	1909.90	1908.88	6.75	−0.487	−1/+1	90.0	67.0	80.0
24 ^e^	LLLLMLGGGLVGGLLGGGGKGGG	1966.24	1965.14	8.75	+1.209	0/+1	92.0	57.0	76.0
25 ^e^	PFLLGVGGLLGGSVGGGGGGGGAPL	2023.14	2022.09	5.96	+0.912	0/0	69.0	32.0	38.0
26 ^e^	GMVVKHCSAPLDSFAEFAGA	2036.93	2035.95	5.32	+0.565	−2/+1	42.0	20.0	26.0
27 ^d^	LPFLGLVGGLLGGSVGGGGGGGGPAL	2136.20	2135.17	5.52	+1.023	0/0	69.1	32.0	38.2
28 ^d^	DVESLPVGGLGGGGGGAGGGGLVGGNLGGGAG	2479.20	2478.21	3.67	+0.353	−2/0	55.0	42.0	30.0

* Peptides’ physicochemical characteristics (isoelectric points (pI), grand average of hydropathicity (GRAVY), and net charge) were determined using the ExPASy ProtParam tool [24]. ^a^ Vassilev et al., 2020 [19]; ^b^ Kostadinova et al., 2018 [15]; ^c^ Beluhova et al., 2022 [25]; ^d^ Dolashki et al., 2020 [18]; ^e^ Topalova et al., 2022 [26]; ^f^ Velkova et al., 2018 [27].

## Data Availability

The authors confirm that the data supporting the findings of this study are available within the article. Row data that support the findings of this study are available from the corresponding author upon reasonable request.
